# Quality of Life (QoL) and Psychosocial Outcomes in Adult Survivors of Unilateral Retinoblastoma (RB) in China

**DOI:** 10.1155/2020/4384251

**Published:** 2020-03-12

**Authors:** Yiyi Feng, Chuandi Zhou, Renbing Jia, Yefei Wang, Xianqun Fan

**Affiliations:** ^1^Department of Ophthalmology, Ninth People's Hospital, Shanghai Jiao Tong University School of Medicine, Shanghai, China; ^2^Shanghai Key Laboratory of Orbital Diseases and Ocular Oncology, Shanghai, China

## Abstract

**Purpose:**

To assess the quality of life (QoL) and the long-term psychosocial outcomes in adult survivors of unilateral retinoblastoma (RB).

**Methods:**

This is a cross-sectional study. Enrolment was offered to all adult survivors of unilateral RB who were treated by enucleation and were followed up in the Ninth People's Hospital of Shanghai Jiao Tong University School of Medicine; noncancer healthy adults served as a control group. All participants completed a series of questionnaires consisting of two aspects, QoL and psychosocial status. The psychosocial outcomes included anxiety, depression, fear of cancer, and satisfaction with facial appearance.

**Results:**

A total of 66 RB survivors (43.0% male) and 66 healthy adults (33.3% male) were aged 27.94 ± 7.63 and 29.18 ± 8.37 at the time of the study, respectively. Adult RB survivors did not have significantly higher rates of depression and anxiety compared with the control group, and they experienced a relatively good QoL. RB survivors were more likely to worry about their facial appearance (median, 1.59 [inter-quartile range, IQR, 1.27 to 2.16] v median, 0.36 [IQR, 0.09 to 1.18]; *p* < 0.001). Radiotherapy was not the factor affecting satisfaction with facial appearance (*β*, −0.27 [confidence interval, CI, −0.69 to 0.16]; *p* < 0.001). Radiotherapy was not the factor affecting satisfaction with facial appearance (*p* < 0.001). Radiotherapy was not the factor affecting satisfaction with facial appearance (*p* < 0.001). Radiotherapy was not the factor affecting satisfaction with facial appearance (

**Conclusions:**

Unilateral RB survivors are a fairly healthy and resilient group. Most unilateral RB survivors experience a relatively good QoL, and they do not have poorer psychosocial functioning compared with a noncancer sample. Females may need more specific psychosocial care.

## 1. Introduction

Retinoblastoma (RB), the most common intraocular malignant tumour that occurs in children, can be destructive, life-threatening, and can cause blindness. The incidence of RB is similar worldwide at 1 in 15000–20000 live births, which corresponds to approximately 9000 new cases every year [1, 2]. Based on the 2013 population estimates, 43% of the individuals with RB live in Asia, and the prevalence in China was only second to that in India [3]. As modern medicine techniques have advanced, the survival rate is higher than 95%, especially in Europe and America [4–6], and the long-term complications in the treatment of RB and the well-being of RB survivors have received increasingly more attention [7, 8]. Previous studies have demonstrated that most RB survivors experience a relatively good but slightly decreased overall quality of life (QoL) [9] and have good psychosocial functioning [7]. In China, many studies on the management, survival rate, and complications related to RB treatments have been published; however, little is known about the QoL and long-term psychosocial functioning.

Evaluations of QoL enable us to assess RB survivors' perception of the impact of their disease on their physical, mental, and social state. In addition, detailed psychosocial questionnaires can assess different aspects of survivors' mental status. The purpose of our study was to characterize the QoL and the long-term psychosocial outcomes among adult survivors of unilateral RB and to find the factors affecting psychosocial outcomes. The results of this study provide insight into the QoL and the long-term psychosocial outcomes of RB patients in China that may contribute to the development of more specific psychosocial patient care.

## 2. Methods

### 2.1. Participants

This is a cross-sectional study. From February 2018 to October 2019, all eligible unilateral RB survivors, treated by enucleation from 1970 to 2017 and followed up in Ninth People's Hospital of Shanghai Jiao Tong University School of Medicine, were invited to participate in this study. The inclusion criteria were survivors who were diagnosed with unilateral RB and treated by enucleation and survivors aged ≥18 who could accurately understand the content of questionnaires. All survivors were contacted by telephone or e-mail, details of this research were provided to them and appointments were made with interested survivors. On the day of appointment or follow-up, participants provided informed consent and completed the assessment by e-mail or telephone interview. Healthy adults who received routine eye examination or accompanied family or friend (not RB) for the hospital visit were invited to participate in this study as a control group. Only one adult per family or group was eligible for enrolment. The inclusion criteria were as follows: individuals aged ≥18; individuals able to understand the questionnaires; individuals without organic diseases; and individuals who provided informed consent. This study was conducted in accordance with the tenets of the Declaration of Helsinki, and informed consent was obtained from all participants.

### 2.2. Survey

The sociodemographic information was obtained by personal communication, and the medical records of each patient were reviewed.

The QoL was measured by the Chinese version of the Medical Outcome Study 36-item Short Form (SF-36) [10]. This questionnaire contains eight separate subscales representing physical functioning (PF), role-physical (RP), bodily pain (BP), general health (GH), vitality (VT), social functioning (SF), role-emotional (RE), and mental health (MH). The raw scores of each subscale can be transformed to standardized scores from 0 to 100, with a higher score reflecting a better QoL.

The Hospital Anxiety and Depression Scale (HAD) and Fear of Progression Questionnaire-Short Form (FoP-Q-SF) were used to measure the three aspects of the psychosocial outcomes of the survivors. The HAD scale is a 14-item self-report measure that contains two subscales focusing on anxiety and depression. Each item is scored from 0 to 3, and higher scores are associated with higher levels of anxiety and depression. A score of 8 commonly is used as the cutoff for anxiety and depression. Fear of cancer was assessed by using FoP-Q-SF [11]. It is a 5-point Likert scale, and each item is scored from 1 (never) to 5 (always), with higher scores indicating greater fear. Participants with scores greater than 34 are thought to have psychological dysfunction. The Negative Physical Self Scale-Facial Appearance Concern (NPSS-F) was used to assess satisfaction with facial appearance [12]. This scale contains three separate subscales representing cognition-affect (CA), behavior (B) and projection (P). It has 11 items, and each item is scored from 0 (never) to 4 (always). The final scores of this scale is the average of the total scores of all items, with higher scores reflecting lower levels of satisfaction. A score of 2 commonly is used as the cutoff for satisfaction.

### 2.3. Statistical Analysis

Analyses were carried out using the software package SPSS 19.0 for Windows. Differences in the characteristics between groups were examined by the Mann–Whitney *U* test or two-sample *t*-test for continuous variables according to the distribution of the data. Ordinal categorical variables were analysed by the Mann–Whitney *U* test. Also, the chi-square test or Fisher's exact test were used to examine the differences between groups for unordered categorical variables. The Mann–Whitney *U* test or two-sample *t*-test were used to compare the scores of QoL and psychosocial outcomes according to the distribution of the data. Demographics, QoL scores, and psychosocial outcomes were summarized using means, standard deviations (SD), medians, and interquartile ranges (IQR) or as proportions, frequencies, and percentages when appropriate. We performed univariate analysis and multivariate regression analysis to screen variables associated with FoP-Q-SF scores and NPSS-F scores. In all tests, two-tailed *p* values of less than 0.05 were deemed to be statistically significant.

## 3. Results

### 3.1. Characteristics of RB Survivors and the Control Group

In total, 139 adult survivors were eligible for this study. However, 42 (30.2%) survivors were lost to follow-up. Of the remaining 97 survivors who were approached, 31 (32.0%) survivors refused to participate and 66 (68.0%) survivors agreed to participate in our study (see [Supplementary-material supplementary-material-1] in the Supplementary Material). In addition, 66 healthy participants were enrolled in this study as the control group. Among the 66 RB survivors, 31 (43.0%) were male (Table 1). The mean age at the time of the study was 27.94 years (SD, 7.63 years). Also, the mean age at diagnosis was 3.15 (SD, 4.43 years). Forty-one (62.1%) were treated with postoperative radiotherapy. Eleven (16.7%) were treated with chemotherapy. Implantation of orbital implant was performed in 52 (78.8%) RB survivors. Most of the survivors had post high school education (56.1%), were living with a family member or friend (72.7%), and were single (57.6%); 12 (18.2%) thought that they had a heavy financial burden. Among the 66 individuals in the control group, 22 (33.3%) were male. The mean age at the time of the study was 29.18 years (SD, 8.37 years). Most of the individuals had post high school education (59.1%), were living with a family member or friend (75.8%), and were married (51.5%); and 9 (13.6%) thought that they had a heavy financial burden. Compared with the RB survivors, there was no significant difference between groups in any of the sociodemographic variables.

### 3.2. RB Survivors Compared with the Control Group

In the SF-36 scale, there were significant differences between RB survivors and the control group in the subscale scores of PF, BP, and RE (Table 2). Interestingly, RB survivors seemed to have more ability to perform physical activities without limitations and more capacity to perform daily activities without interference from emotional problems. Also, they had less pain which might interfere with daily activities. In the HAD scale, the scores in the subscale of anxiety showed significant difference (Table 3). RB survivors had less anxiety compared with the control group (*p* = 0.045). However, the percentage of participants with a score of 8 or higher was 14.1% of the RB survivors and 27.3% of the control group, which had no significant difference (*p* = 0.063). In the subscale of depression, the scores and the sickness rate did not show significant differences. According to the NPSS-F, RB survivors were significantly more likely to worry about their facial appearance than the individuals in the control group, and the scores of RB survivors were much higher than those of the control group (median, 1.59 [IQR, 1.27 to 2.16] v median, 0.36 [IQR, 0.09 to 1.18]; *p* < 0.001). Degree of satisfaction was much lower among RB survivors compared with the control group, and 20 (31.3%) RB survivors were unsatisfied with facial appearance (*p* < 0.001) (Table 4).

### 3.3. RB Survivors with Radiotherapy versus Those without Radiotherapy

Between RB survivors with radiotherapy and those without radiotherapy, there were no significant differences in any of the SF-36 subscale scores (Table 2). Analyses on the HAD scores indicated that the differences between groups were not significant (Table 3).

### 3.4. Univariate Analyses and Multivariate Regression Analyses of Factors Affecting FoP-Q-SF Scores and NPSS-F Scores

Among RB survivors, 9 (14.1%) had psychological dysfunction according to the FoP-Q-SF scores (Table 5). The univariate analyses of the effects of different independent variables on the scores of FoP-Q-SF and NPSS-F showed that the influence of sex was statistically significant (Table 6). Variables with *p* < 0.10 in univariate analyses were included in the multivariate model. However, the results indicated that only sex was the influencing factor. Females had higher scores than the males did (median, 28.50 [IQR, 23.75 to 35.00] v median, 26.00 [IQR, 17.50 to 29.25]; *p* = 0.031) in the FoP-Q-SF. 23.5% of females had psychological dysfunction (*p* = 0.021) (Table 5). In the NPSS-F scale, females were significantly more likely to worry about their appearance (mean, 1.88 [SD, 0.84] v mean, 1.46 [SD, 0.77]; *p* = 0.041), especially in the CA subscale (*p* = 0.039) (Table 4). However, the satisfaction rate between females and males had no significant difference.

## 4. Discussion

To our knowledge, this is the first report to focus on the QoL and the long-term psychosocial outcomes in Chinese adult RB survivors. In this study, we demonstrate that most unilateral RB survivors experience a relatively good QoL, and they do not have poorer psychosocial functioning.

Regarding adult RB survivors, the Dutch study showed that they experienced a relatively good overall QoL, except for mental health outcomes, which were slightly worse among RB survivors when compared with a reference group [9]. Another study of 69 adult RB survivors in USA showed that adult survivors of RB demonstrated few cognitive or social attainment deficits decades following diagnosis and treatment [13]. With respect to psychosocial conditions, a study of 470 adult RB survivors showed that most RB survivors do not have poorer psychosocial functioning and some of their findings indicated that RB survivors were doing better psychosocially compared with a noncancer sample [7]. The same conclusion was also drawn by our study. In our study, the results suggest that unilateral RB survivors have a better QoL and they exhibit good psychosocial conditions compared with the control group, which is not consistent with our normal perception. All these studies describe adult RB survivors as a fairly healthy and resilient group. Posttraumatic growth (PTG) may have played an important role among these RB survivors [14, 15]. In our study, only unilateral RB survivors were included, we did not explore the difference between bilateral RB survivors and unilateral RB survivors. A study found that history of bilateral disease was associated with inferior overall QoL; however, bilateral and unilateral RB survivors seem similar with respect to their psychological symptoms [7, 16].

Regarding children with RB, most of the previous studies conducted in developed countries have investigated QoL and also found that children with RB experience a relatively good QoL [17, 18]. However, the studies in India and China suggest that the QoL of children with RB is significantly lower than that of normal children [19, 20]. A lower age at diagnosis seemed to be the key factor that determined a higher QoL. The younger survivors may not have memories of treatment-related stress and morbidities and thus have better adjustment than do older survivors, which may be the reason for this observation [19]. Moreover, early discovery of the disease can improve ocular salvage rate and vision, which may improve QoL. The differences in the results between developed and developing countries may be associated with different medical and social support systems, which are far from perfect in developing countries. Therefore, it is important to expose the current situation of RB survivors not only with respect to management of the disease but also with respect to their well-being, which may contribute to the development of medical and social support systems.

Our study also showed that females were more likely to have the fear of being influenced by the disease compared with males (See [Supplementary-material supplementary-material-1] in the Supplementary Material). Also, females were more concerned about their appearance, especially on the cognition-affect, perhaps highlighting a greater need for long-term psychosocial counselling.

Some limitations of the present study should be addressed. Although this is the first study to assess the QoL and psychosocial status of Chinese adult RB survivors, only unilateral RB survivors were included. Secondly, some survivors were lost to follow-up, which may cause bias. Besides, radiotherapy could increase the risk of socket contracture [21], which might decrease the satisfaction with facial appearance. However, in our study, prior radiotherapy exposure was not associated with inferior QoL and poorer psychosocial functioning. This could be due to the fact that most RB survivors in our study underwent radiotherapy, which led to a low statistical power. In addition, our study did not objectively evaluate the appearance of RB survivors, such as orbital volume, asymmetry, and degree of soft tissue atrophy, which resulted in the failure of further analysing the impacts of appearance defects caused by enucleation and radiotherapy on the QoL and the psychosocial outcomes.

## 5. Conclusions

In conclusion, our study indicates that adult survivors of unilateral RB in China generally experience a relatively good QoL compared with a control group. They exhibited good psychosocial conditions but were generally dissatisfied with their facial appearance. Also, females may need more specific psychosocial care.

## Figures and Tables

**Table 1:** Characteristics of RB survivors and the control group.

Variable	All RB survivors (*n* = 66)	Survivors with radiotherapy (*n* = 41)	Survivors without radiotherapy (*n* = 25)	Control group (*n* = 66)	*p*	*p* ^a^
*Age at study*					0.400	0.056
Mean (SD), years	27.94 (7.63)	26.24 (6.16)	30.72 (9.03)	29.18 (8.37)		
Median (IQR), years	25.50 (22.00–32.25)	25.00 (21.50–30.50)	29.00 (23.00–38.50)	26.00 (22.00–34.00)		
*Age at diagnosis*					NA	0.042
Mean (SD), years	3.15 (4.43)	2.85 (1.48)	3.64 (7.01)	NA		
Median (IQR), years	2.00 (1.00–4.00)	3.00 (2.00–4.00)	1.00 (1.00–3.00)	NA		
*Sex, n (%)*					0.110	0.376
Female	35 (57.0%)	20 (48.8%)	15 (60.0%)	44 (66.7%)		
Male	31 (43.0%)	21 (51.2%)	10 (40.0%)	22 (33.3%)		
*Education, n (%)*					0.829	0.573
Completed junior high school or less	8 (12.1%)	6 (14.6%)	2 (8.0%)	14 (21.2%)		
Completed high school	20 (30.3%)	12 (29.3%)	8 (32.0%)	13 (19.7%)		
Completed university or more	37 (56.1%)	22 (53.7%)	15 (60.0%)	39 (59.1%)		
Unknown/missing	1 (1.5%)	1 (2.4%)	0	0		
*Marital status, n (%)*					0.295	0.474
Single	38 (57.6%)	25 (61.0%)	13 (52.0%)	32 (48.5%)		
Married	28 (42.4%)	16 (39.0%)	12 (48.0%)	34 (51.5%)		
*Financial burden, n (%)*					0.415	0.874
Light	32 (48.5%)	20 (48.8%)	12 (48.0%)	24 (36.4%)		
Medium	22 (33.3%)	14 (34.1%)	8 (32.0%)	33 (50.0%)		
Heavy	12 (18.2%)	7 (17.1%)	5 (20.0%)	9 (13.6%)		
*Living with family or friend, n (%)*					0.691	0.641
Yes	48 (72.7%)	12 (29.3%)	6 (24.0%)	50 (75.8%)		
No	18 (27.3%)	29 (70.7%)	19 (76.0%)	16 (24.2%)		
*Orbital implant, n (%)*						0.419
Yes	52 (78.8%)	31 (75.6%)	21 (84.0%)	NA	NA	
No	14 (21.2%)	10 (24.4%)	4 (16.0%)	NA	NA	
*Chemotherapy, n (%)*						0.308
Yes	11 (16.7%)	5 (12.2%)	6 (24.0%)	NA	NA	
No	55 (83.3%)	36 (87.8%)	19 (76.0%)	NA	NA	

SD, standard deviation; IQR, interquartile range; NA, not applicable; RB, retinoblastoma. *p*^a^, *p* value compared characteristic differences between RB survivors with or without radiotherapy. *p*, *p* value compared characteristic differences between RB survivors and the control group.

**Table 2:** RB survivors versus the control group and RB survivors with radiotherapy versus RB survivors without radiotherapy in the SF-36.

Domain median (IQR)	All RB survivors (*n* = 66)	Survivors with radiotherapy (*n* = 41)	Survivors without radiotherapy (*n* = 25)	Control group (*n* = 66)	*p*	*p* ^a^	*p* ^a*∗*^
Physical functioning	100.00 (95.00–100.00)	100.00 (100.00–100.00)	100.00 (95.00–100.00)	95.00 (90.00–100.00)	0.001	0.081	0.170
Role-physical	87.50 (50.00–100.00)	100.00 (75.00–100.00)	75.00 (37.50–100.00)	100.00 (50.00–100.00)	0.089	0.200	0.321
Bodily pain	100.00 (72.00–100.00)	100.00 (72.00–100.00)	100.00 (72.00–100.00)	74.00 (69.50–100.00)	0.011	0.572	0.703
General health	75.00 (55.00–87.00)	77.00 (57.00–87.00)	62.00 (52.00–82.00)	71.00 (52.00–85.50)	0.529	0.129	0.105
Vitality	75.00 (65.00–80.00)	80.00 (70.00–85.00)	75.00 (60.00–80.00)	70.00 (55.00–80.00)	0.076	0.115	0.152
Social functioning	90.00 (76.88–100.00)	90.00 (71.25–100.00)	87.50 (77.50–100.00)	87.50 (77.50–100.00)	0.531	0.629	0.891
Role-emotional	100.00 (66.67–100.00)	100.00 (66.67–100.00)	100.00 (33.33–100.00)	66.67 (0.00–100.00)	0.002	0.268	0.350
Mental health	80.00 (64.00–84.00)	80.00 (72.00–84.00)	80.00 (62.00–84.00)	72.00 (60.00–84.00)	0.119	0.436	0.278

*p* ^a*∗*^, *p* values were analysed after adjusting for age at diagnosis.

**Table 3:** RB survivors versus the control group and RB survivors with radiotherapy versus RB survivors without radiotherapy in the HAD.

	All RB survivors (*n* = 64)	With radiotherapy (*n* = 39)	Without radiotherapy (*n* = 25)	Control group (*n* = 66)	*p*	*p* ^a^	*p* ^a*∗*^
Anxiety, median (IQR)	4.00 (2.00–6.00)	4.00 (1.00–6.00)	4.00 (2.50–6.00)	5.00 (2.75–8.25)	0.045	0.406	0.534
Positive, *n* (%)	9 (14.1%)	5 (12.8%)	4 (16.0%)	18 (27.3%)	0.063	0.728	0.993
Depression, median (IQR)	2.00 (1.00–5.00)	2.00 (1.00–5.00)	2.00 (1.00–6.00)	4.00 (1.75–7.00)	0.080	0.713	0.957
Positive, *n* (%)	6 (9.4%)	4 (10.3%)	2 (8.0%)	12 (18.2%)	0.146	1.000	0.521

Two patients were excluded because of missing data. *p*^a*∗*^, *p* values were analysed after adjusting for age at diagnosis.

**Table 4:** RB survivors versus the control group and male versus female in the NPSS-F

	All RB survivors (*n* = 64)	Male (*n* = 30)	Female (*n* = 34)	Control group (*n* = 66)	*p*	*p* ^*∗*^
Total score, median (IQR)/mean (SD)	1.59 (1.27–2.16)	1.46 (0.77)	1.88 (0.84)	0.36 (0.09–1.18)	<0.001	0.041
Unsatisfied, *n* (%)	20 (31.3%)	8 (26.7%)	12 (35.3%)	3 (4.5%)	<0.001	0.461
Cognition-affect (CA), median (IQR)	1.63 (0.50–2.25)	1.38 (0.50–2.00)	2.00 (1.00–2.56)	0.50 (0.00–1.00)	<0.001	0.039
Behavior (B), median (IQR)	3.00 (2.00–3.33)	2.50 (1.00–3.50)	3.00 (2.00–3.33)	0.67 (0.00–1.67)	<0.001	0.475
Projection (P), median (IQR)	1.00 (0.25–1.75)	1.00 (0.00–1.50)	1.13 (0.44–2.00)	0.25 (0.00–1.00)	<0.001	0.282

Two patients were excluded because of missing data. *p*^*∗*^, *p* value compared NPSS-F scores between males and females.

**Table 5:** Male versus female in the FoP-Q-SF.

	All RB survivors (*n* = 64)	Male (*n* = 30)	Female (*n* = 34)	*p*
Total score, mean (SD)	27.14 (8.39)	24.37 (6.92)	29.59 (8.89)	0.031
Total score, median (IQR)	27.00 (21.25–32.00)	26.00 (17.50–29.25)	28.50 (23.75–35.00)	
>34, *n* (%)	9 (14.1%)	1 (3.3%)	8 (23.5%)	0.021

*p*, *p* value compared FoP-Q-SF scores between males and females.

**Table 6:** Univariate analysis and multivariate regression analysis of factors affecting FoP-Q-SF scores and NPSS-F scores.

Variable	FoP-Q-SF				NPSS-F			
	Univariate analysis		Multivariable analysis		Univariate analysis		Multivariable analysis	
	*β* (95% CI)	*p*	*β* (95% CI)	*p*	*β* (95% CI)	*p*	*β* (95% CI)	*p*
Age at study	−0.02 (−0.32 to 0.27)	0.866	NA	NA	−0.01 (−0.04 to 0.02)	0.631	NA	NA
Age at diagnosis	0.18 (−0.29 to 0.65)	0.450	NA	NA	−0.01 (−0.05 to 0.04)	0.845	NA	NA
Education	0.25 (−2.74 to 3.25)	0.866	NA	NA	−0.14 (−0.44 to 0.15)	0.335	NA	NA
Financial burden	2.30 (−0.39 to 4.99)	0.092	1.96 (−0.64 to 4.56)	0.137	0.21 (−0.06 to 0.48)	0.122	NA	NA
Sex	5.22 (1.20 to 9.24)	0.012	4.90 (0.90 to 8.90)	0.017	0.42 (0.02 to 0.83)	0.041	0.41 (0.01 to 0.80)	0.045
Marital status	−1.60 (−5.88 to 2.68)	0.459	NA	NA	−0.22 (−0.65 to 0.20)	0.293	NA	NA
Living with family or friend	1.97 (−2.70 to 6.64)	0.402	NA	NA	0.27 (−0.19 to 0.73)	0.238	NA	NA
Orbital implant	−0.18 (−5.29 to 4.93)	0.944	NA	NA	0.47 (−0.03 to 0.96)	0.063	0.45 (−0.04 to 0.93)	0.069
Chemotherapy	−1.37 (−6.96 to 4.22)	0.627	NA	NA	0.12 (−0.44 to 0.67)	0.680	NA	NA
Radiotherapy	0.75 (−3.57 to 5.08)	0.729	NA	NA	−0.27 (−0.69 to 0.16)	0.214	NA	NA

A linear regression model was used to examine the association between scale scores and the independent variables. *β*, the regression coefficient; CI, confidence interval. Variables with *p* > 0.10 in univariate analysis were not included in the multivariate model and are indicated by NA in the table.

## Data Availability

The data used to support the findings of this study are available from the corresponding author upon request.

## Abbreviations

QoL: Quality of life
RB: Retinoblastoma
SD: Standard deviation
SF-36: Medical outcome study 36-item short form
PF: Physical functioning
RP: Role-physical
BP: Bodily pain
GH: General health
SF: Social functioning
RE: Role-emotional
MH: Mental health
HAD: Hospital anxiety and depression scale
FoP-Q-SF: Fear of progression questionnaire-short form
NPSS-F: Negative physical self scale-facial appearance concern
CA: Cognition-affect
PTG: Posttraumatic growth
NA: Not applicable
IQR: Interquartile range
CI: Confidence interval.

## Supplementary Materials

Supplementary Materials Figure S1: recruitment and participation for retinoblastoma survivors. Table S1: male versus female in the FoP-Q-SF.
