# Supplementary material for: Quality of Life (QoL) and Psychosocial Outcomes in Adult Survivors of Unilateral Retinoblastoma (RB) in China
Source: J Ophthalmol. 2020 Mar 12;2020:4384251. doi: 10.1155/2020/4384251 (PMC7125462; doi:10.1155/2020/4384251)
Supplement: Supplementary Materials — Figure S1: recruitment and participation for retinoblastoma survivors. Table S1: male versus female in the FoP-Q-SF. [file 4384251.f1.pdf]

Supplementary Materials

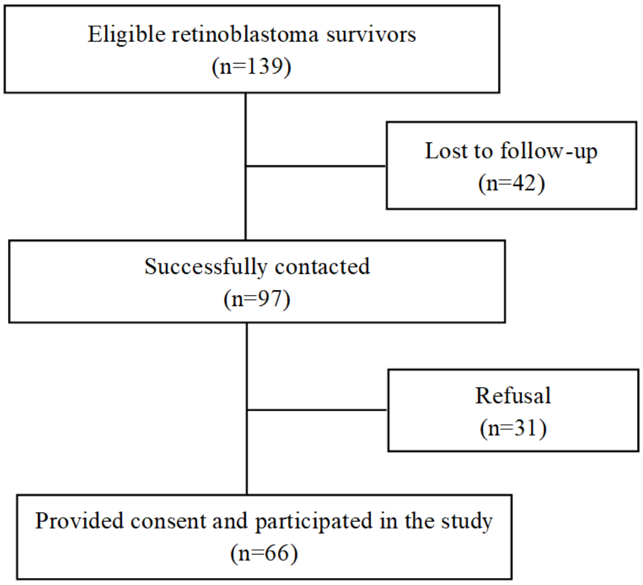

Figure S1: Recruitment and participation for retinoblastoma survivors.

Table S1: Male versus female in the FoP-Q-SF

Item	All RB survivors (n=64)	Male (n=30)	Female (n=34)	*p*
Item 1 Being afraid of disease progression				0.007
Never/ Few/ Sometime	56 (87.5%)	30 (100.0%)	26 (76.5%)	
Often/ Always	8 (12.5%)	0	8 (23.5%)	
Item 2 Being nervous prior to doctor's appointments or periodic examinations				0.364
Never/ Few/ Sometime	52 (81.3%)	24 (80.0%)	28 (82.4%)	
Often/ Always	12 (18.7%)	6 (20.0%)	6 (17.6%)	
Item 3 Being afraid of pain				0.886
Never/ Few/ Sometime	54 (84.4%)	27 (90.0%)	27 (79.4%)	
Often/ Always	10 (15.6%)	3 (10.0%)	7 (20.6%)	
Item 4 Being afraid of becoming less productive at work				0.081
Never/ Few/ Sometime	57 (89.1%)	29 (96.7%)	28 (82.4%)	
Often/ Always	7 (10.9%)	1 (3.3%)	6 (17.6%)	
Item 5 Having physical symptoms (e.g., rapid heartbeat, stomachache)				0.047
Never/ Few/ Sometime	62 (96.9%)	29 (96.7%)	33 (97.1%)	
Often/ Always	2 (3.1%)	1 (3.3%)	1 (2.9%)	
Item 6 Being afraid by the possibility that the children could contract the disease				0.058
Never/ Few/ Sometime	48 (75.0%)	27 (90.0%)	21 (61.8%)	
Often/ Always	16 (25.0%)	3 (10.0%)	13 (38.2%)	
Item 7 Being afraid of relying on strangers for activities of daily living				0.007
Never/ Few/ Sometime	57 (89.1%)	30 (100.0%)	27 (79.4%)	
Often/ Always	7 (10.9%)	0	7 (20.6%)	
Item 8 Being afraid of no longer being able to pursue hobbies				0.080
Never/ Few/ Sometime	60 (93.7%)	29 (96.7%)	31 (91.2%)	
Often/ Always	4 (6.3%)	1 (3.3%)	3 (8.8%)	
Item 9 Being afraid of severe medical treatments in course of illness				0.176
Never/ Few/ Sometime	53 (82.8%)	26 (86.7%)	27 (79.4%)	
Often/ Always	11 (17.2%)	4 (13.3%)	7 (20.6%)	
Item 10 Worrying that medications could damage the body				0.231
Never/ Few/ Sometime	53 (82.8%)	27 (90.0%)	26 (76.5%)	
Often/ Always	11 (17.2%)	3 (10.0%)	8 (23.5%)	
Item 11 Worrying what will become of family if something happens to me				0.565
Never/ Few/ Sometime	52 (81.3%)	25 (83.3%)	27 (79.4%)	
Often/ Always	12 (18.8%)	5 (16.7%)	7 (20.6%)	
Item 12 Being afraid of not being able to work anymore				0.068
Never/ Few/ Sometime	49 (76.6%)	26 (86.7%)	23 (67.6%)	
Often/ Always	15 (23.4%)	4 (13.3%)	11 (32.4%)	
